# What Was Helpful Questionnaire (WHQ): Psychometric Properties of a Novel Tool Designed to Capture Parental Perceived Helpfulness of Interventions in Children Requiring Mental Health Inpatient Care

**DOI:** 10.3389/fpsyt.2019.00080

**Published:** 2019-02-26

**Authors:** Ifigeneia Mourelatou, Jorge Gaete, Sandra Fewings, Oona Hickie, Marinos Kyriakopoulos

**Affiliations:** ^1^National and Specialist Acorn Lodge Inpatient Children's Unit, South London and Mausdley NHS Foundation Trust, Bethlem Royal Hospital, Beckenham, United Kingdom; ^2^Department of Public Health and Epidemiology, Faculty of Medicine, Universidad de los Andes, Santiago, Chile; ^3^Department of Child and Adolescent Psychiatry, Institute of Psychiatry, Psychology and Neuroscience, King's College London, London, United Kingdom; ^4^Department of Psychiatry, Icahn School of Medicine at Mount Sinai, New York, NY, United States

**Keywords:** children, inpatient care, helpfulness, mental health, validation

## Abstract

**Background:** Children in mental health inpatient care require multiple treatments. There is not a comprehensive instrument to assess perceived helpfulness of this combination of interventions.

**Aims:** To develop and evaluate the psychometric properties of the What was Helpful Questionnaire (WHQ), a tool designed to capture parental perceived helpfulness of the multidimensional management approach used in inpatient children's units.

**Methods:** A total of 73 inpatients and their families were included in this study. The WHQ consists of six items exploring the perceived helpfulness of different aspects of care. Demographic and clinical variables were collected on admission and discharge. An exploratory factor analysis using polychoric correlations was performed to assess the item structure of the scale and the Cronbach's alpha coefficient was used for internal reliability. Associations were assessed using regressions models.

**Results:** WHQ is a unidimensional scale with an internal reliability of 0.77. No associations were identified between WHQ total score and age, gender, and Children's Global Assessment Scale scores change. A strong relationship between the WHQ total score and parental Acorn Satisfaction Questionnaire total score was found.

**Conclusions:** Results add evidence for the validity and the reliability of the WHQ to measure parental perceived helpfulness of interventions offered in inpatient children's units.

## Introduction

The prevalence of psychopathology among children and adolescents is high (1) and its impact extends to adulthood affecting academic outcomes (2), increasing the risk of other mental health problems (2, 3) and reducing life satisfaction (4). There is some agreement that 1 in 5 children will suffer from a psychiatric condition before entering adulthood (5).

The clear majority of children and adolescents diagnosed with mental health conditions are treated in outpatient services. However, many young people have severe disorders difficult to manage in community settings and require inpatient input. In the United Kingdom (UK), most hospital mental health beds for under-18s are dedicated to adolescents as their clinical presentation is more frequently, compared to younger children, associated with challenges that need this higher intensity of care (6). Children's inpatient mental health units are a particularly specialized National Health Service provision for young people up to the age of 12 years with complex or/and severe mental health problems. These units offer comprehensive and individualized assessment and multidimensional treatment, including specialist educational input, that can lead to significant positive changes for both children and their families (7, 8). The multidimensional treatment includes behavioral management, psychological and family interventions and, commonly, medication.

There are many reasons behind admission in an inpatient service instead of outpatient treatment, as well as dilemmas regarding inpatient care. Commonly, in the case of conditions associated with significant clinical risk, and particularly retractable or even deteriorating symptoms despite the most intensive outpatient treatment, inpatient care can be offered as an alternative. Admission in an inpatient unit allows detailed assessment of complex presentations in a controlled setting, comprehensive observations, and the development of effective strategies to assist the child and their family manage their difficulties after discharge (9, 10). Importantly, it allows the introduction of a new treatment, such as medication, to be observed (10) and continuous risk management approaches to be implemented (e.g., in cases of self-harm or aggression) (9). Inpatient units can help children improve in their functioning, socialization and academic skills and can have a major impact on young people, who might have a history of difficulties regarding their social adaptation or/and school failure. The individualized assessment and intensive specialist treatment can lead to more effective use of other services in the future (9).

However, there are certain challenges that need to also be taken into consideration. Inpatient admission can have a negative impact on the family or the child due to the dislocation and loss of support from a familiar environment and peer group, especially in cases where units are distant from the children's homes. This situation is not uncommon, considering the aforementioned lack of capacity in inpatient children's mental health services and the uneven geographical distribution of existing units. Other concerns include potential “learning” of new symptoms from other young people as well as institutionalization if the admission is prolonged with subsequent dependency (9, 10).

Today standards of care require the assessment of patient reported outcomes (PROs), that is, outcomes directly reported by patients without further interpretation by clinicians, but also the incorporation of valid and reliable instruments or tools to evaluate patient perception with patient reported outcome measures (PROMs) (11). For example, it is mandatory in United Kingdom (UK), since 2009, to have PROMS for many health conditions (11). In recent years, clinical care has incorporated instruments or tools to measure functional status, health related quality of life, health behaviors (11, 12), patient satisfaction (13) and patient—reported experience measures (PREMs) (14–16).

Measures of patient's satisfaction have acquired significant importance in health care, particularly in treatment outcomes and intervention engagement (17–21) and constitute an indispensable aspect of direct evaluation of health care services and providers (22). Satisfaction is a broad multi-dimensional construct including several aspects of care: access, financial aspects, availability of resources, continuity of care, technical quality, interpersonal manners, and overall satisfaction (22). This evaluation is influenced not only by the actual experience of patients with services (e.g., waiting time in minutes) but also by their expectations (e.g., preferences for long or short office times). Several studies have explored factors related to high satisfaction (23–29) and low satisfaction with psychiatric services (30–33). Moreover, patient's satisfaction measurements have been included in different health systems, for example, United States, Canada, and UK (34), because they help to evaluate the quality of care, quality of services, patient experience, and other patient—based health outcome studies (35).

However, much less attention has received the assessment of the perceived helpfulness of different treatments by patients or their caregivers. Similarly to patient satisfaction, perceived helpfulness is a subjective evaluation; however, helpfulness seems to be a more specific construct. Perceived helpfulness is the perception of the quality of the service or assistance provided by others (people or technological advances) (36). In the medical field, most of the studies have linked helpfulness to treatments. There are few studies exploring psychometric features of questionnaires exploring treatment helpfulness (37, 38), even though the construct has been included in several services and treatment evaluations. For example, perceived helpfulness is likely to facilitate families' involvement, lead to more effective interventions, and improved outcome measures. Lee and colleagues (2010) reported that patients perceived the care and treatment to be helpful after experiencing improvements or changes in their physical (e.g., gastric relief, improved blood circulation, feeling more energetic), cognitive (e.g., reduction of anxiety, sense of calmness, security, self-awareness, hopefulness), or interpersonal (e.g., reduction of interpersonal conflicts) functioning (39). There are few studies exploring the factors influencing perceived helpfulness, but it appears that etiological information (attributed causes of mental health problems), past experiences, nature of treatments and expectations are important (40–42). Moreover, family and patients may have different perceptions of what is helpful. For example, family-based treatment for adolescent eating disorder was perceived as helpful; however, patients seemed to prioritize cognitive in comparation to physical improvements (43).

The majority of the studies exploring perceived helpfulness refers to treatment or services evaluations. For example, a study compared the patients' perceived helpfulness of depression treatment provided by general medical providers (GMPs) and specialty mental health providers (SMHPs) (44). It found that adults who received depression care from an SMHP or a combination of GMPs and SMHPs considered treatment for depression more helpful than adults who received care from a GMP. These findings highlight the importance of type of provider for mental health problems (44). A recent study compared different self-management strategies to deal with depression, finding that not always the most helpful strategies as they were perceived by the patients (completing the treatment, leaving the house regularly) were the most used (45). Rosemblat et al. studied the frequency of use and perceived helpfulness of less structured interventions for bipolar and unipolar depression (46). Wellness strategies, such as listening to music or having adequate sleep, were reported to be helpful and they were associated with perceived treatment effectiveness and greater subjective helpfulness of medication, psychotherapy, and peer support groups (46). In another study on depression among adolescents, counseling and medication were compared in regards to their helpfulness, finding that 32% to 47% of adolescents in the general population perceived depression treatment as extremely or a lot helpful which was lower than the response rate in clinical trials (47). Additionally, adolescents found that medication was more helpful than counseling (47). Regarding treatment adherence, perceived helpfulness may have a role. For example, in a study among 45 participants with schizophrenia spectrum disorders, perceived helpfulness of the previous treating psychiatrist and of previous medication and feeling insufficiently informed about medication significantly predicted medication discontinuation (48). In another study among children and young people with juvenile idiopathic arthritis, the helpfulness of the medication was associated with high adherence (49). Some studies had also examined the parental role of treatment adherence using helpfulness as an explanatory variable. For example, among children and adolescents with psychiatric conditions, medication adherence was greater in children and mothers when mothers felt that the medication helped to reduce the symptoms (50). Similarly, among patients with juvenile idiopathic arthritis factors associated with higher perceived adherence to medications included perceived helpfulness of medications and lower disease severity (51). All the above examples showed how the perceived helpfulness assessment has contributed in the evaluation of service providers, as well as different treatments, and their association to clinical outcomes and treatment adherence. Studying this construct may also help to personalize mental health care and treatments, which is one of the main challenges for the future (52).

There are, already, different tools to assess treatment progress among children and adolescents receiving support in inpatient facilities. For example, symptomatology can be assessed using parent reported symptoms questionnaires such as the Strengths and Difficulties Questionnaire (SDQ) (53), or clinician reported functionality questionnaires such as the Children's Global Assessment Scale (CGAS) (54) or the Health of the Nation Outcome Scales for Children and Adolescents (HoNOSCA) (55). There are also instruments used to assess client satisfaction regarding the care provided by mental health services, such as Acorn Satisfaction Questionnaire (7). So far, there has been no comprehensive tool to assess the caregivers' perceived helpfulness of different treatments offered in an inpatient mental health care service whether alone or in combination.

The aim of the current study was to develop and evaluate the psychometric properties of the *What was Helpful Questionnaire (WHQ)*, a tool designed to capture parental perceived helpfulness of the multidimensional management approach used in inpatient mental health children's units. Given the high level of integration of different treatment approaches in this sample, we hypothesized that WHQ would assess one latent factor measuring parental perceived helpfulness.

## Materials and Methods

### Setting and Participants

Acorn Lodge provides inpatient assessment and treatment for children aged up to 12 years with severe and complex disorders. These include children with neuropsychiatric disorders such as autism spectrum disorders (ASD) and hyperkinetic disorders (HD), depression, very early onset psychosis and bipolar affective disorder, obsessive compulsive disorder, eating disorders, stress-related disorders, severe encopresis and complicated diagnostic conditions. The service is one of only eight child inpatient units in the UK. It is open seven days a week and offers planned and emergency admissions, including out of hours.

The members of the multidisciplinary team are experienced staff from a variety of professional backgrounds including specialist nurses, a psychologist, a social worker, a family therapist, an occupational therapist and child and adolescent psychiatrists. The ward runs a behavioral model supplemented by other treatment modalities including Cognitive Behavioral Therapy (CBT), family therapy, parenting work, Occupational Therapy (OT) (sometimes working with the child and mother in an action form of therapy, sometimes drama therapy) and pharmacotherapy. The Unit is supported by the Bethlem and Maudsley Hospital School and highly developed educational provision where the children are offered remedial education when appropriate. The school liaises closely with the child's school of origin and with the relevant Educational Authority to help plan appropriate resources for the child's future educational needs. This planning can require funding through complex panel procedures in local authorities. Close liaison with community agencies forms an integral part of the unit's work.

Parents are strongly involved in all aspects of treatment, where their knowledge of their children is valued and their opinions welcome, and participate in family assessments and therapy. Support groups and counseling are also offered for parents and siblings. Parents and family members visit and take children out of the unit as much as possible depending on the children's individual needs. The WHQ was designed in an attempt to capture parental perceived helpfulness of these complex interventions in a valid and reliable way.

### Sample

All 73 inpatients discharged from Acorn Lodge from December 2013 to December 2016 were included in this project. All children and their parents are asked to complete a number of outcome and satisfaction measures upon discharge from the unit including the SDQ, CGAS, HONOSCA, ASQ and WHQ as part of their clinical care. The completion of these scales do not take longer than 20 min; however, in few occasions participants will be given as much time as required.

Analysis of the WHQ was part of a wider service evaluation project of anonymized data.

### Measures

#### Socio-Demographic and Clinical Variables

Information on gender, age on admission, ethnicity, family composition, and length of stay (LOS) was collected from the children's clinical notes. We also collected information about receiving medication during the inpatient treatment.

#### Instruments

What was Helpful Questionnaire (WHQ) at discharge: Two of the authors (SF, MK) created the questionnaire, considering different aspects of care that children normally receive on the ward. The questionnaire was subsequently piloted with a group of parents who did not feel any other aspects of care should be added or any of the included aspects should be removed. The final WHQ (Appendix) consisted of 6 items, rating the perceived helpfulness of each aspect of care on a Likert scale (1 = Strongly Disagree to 5 = Strongly Agree) and was completed by parents at the end of a child's admission. The aspects of treatment included in the questionnaire are briefly described in Table 1. The total score of WHQ ranges from 6 to 30. A higher score means higher perceived helpfulness related to the inpatient care the child received.

**Table 1:** Description of aspects of treatment.

**Aspect of treatment**	**Description**
Family work	Family work/therapy is provided by a Family therapist. This therapist assesses and works with the family using a systemic approach.
Work on parenting skills	Parents receive help to build or develop better parenting skills. This work is provided by all members of the multidisciplinary team.
Behavioral work for children on the ward	Behavioral work is provided by all members of the multidisciplinary team and constitute a big part of the input a child receives as part of the ward milieu.
Individual psychological work for children	Individual psychological work for the child is provided by a trained psychologist or a trainee psychologist under supervision. The main approach is cognitive behavioral therapy.
Medication	Medication treatment as part of a comprehensive treatment plan is prescribed by a Consultant Child and Adolescent Psychiatrist and carefully monitored by trained nursing staff.
Care Plans	Carefully designed care plans to assist with the child's day-to-day management are agreed with them and their families. Each child has a care coordinator and a nursing care team to ensure that all aspects of care are covered.

Children's Global Assessment Scale scores (CGAS) on admission and at discharge: (54) The CGAS was originally created as an adaptation of the Global Assessment Scale for adults (56) and designed to reflect the lowest level of functioning for children during a specified time period. It has values from 1, representing the lowest level of functioning, to 100, representing the highest. Scores over 70 represent normal functioning. The CGAS has good interrater reliability (0.84) and test-retest stability (0.85) (54). It has been used extensively in clinical and research settings.

Health of the Nation Outcome Scale for Children and Adolescents (HoNOSCA) on admission and at discharge: (55) The HoNOSCA is a tool used by clinicians to assess clinical change in children and adolescents attending psychiatric clinics. The instrument comprises 15 simple scales measuring behavior, impairment, symptoms, social problems and information problems for those under 18 years of age. The interrater reliability is 0.82 for psychiatric symptoms.

Acorn Satisfaction Questionnaire (ASQ) at discharge: (8) This is a 9-item questionnaire, with seven items responded by parents and two by children, which has been previously used in this population (7). In this study, we only used the report from parents. The total score is the average of the sum of all items. In our sample, the score ranged from 1 to 5, with a mean score of 4.53 (Standard Deviation (SD) = 0.56). The internal reliability in our sample was 0.81.

### Data Analysis

We examined the children's sociodemographic variables as well as the items' psychometric characteristics by using descriptive statistics (i.e., means, standard deviations, frequencies, percentages, skewness, and kurtosis).

There are several methodologies and approaches that can be implemented to add evidence of the validity of an instrument (57). All these approaches, from the analyses of the item structure (construct validity) to the discrimination between subjects (discriminant validity) and the agreement between different measures assessing the same construct (concurrent validity) provide different perspective of the performance and validity of an instrument. Generally, after the creation of a new instrument, the first analysis consists of the exploration of the dimensionality and item structure (communalities between items and a latent factor) of the scale using an exploratory factor analysis (EFA). Therefore, an EFA using polychoric correlations was performed. Polychoric correlations are advised for factor analysis when the distributions of ordinal items are asymmetric with excess of kurtosis or high item- total correlation (58). For factor extraction, we used principal axis factoring which is recommended if the assumption of multivariate normality is not completely fulfilled (59), with promax oblique rotation method which allows the factors to correlate (60). The selection of the number of factors was based on eigenvalues ≥1.0. To select the items to be included in the latent factor, we used the criterion of factor loading ≥0.32, cut off point corresponding to ~10% overlapping variance with the other items in the factor (60, 61). The total score of the questionnaire required that all items were answered; therefore, the analysis included completed data.

The Internal consistency was assessed using Cronbach's alpha coefficient. The final score was calculated using the average score for each item with valid response. Acceptable values of alpha range from 0.7 to 0.95 (62). For comparing groups, alpha between 0.7 and 0.8 is considered as satisfactory; however, a minimum of 0.90 is recommended for clinical applications (63).

We explored associations between perceived helpfulness and gender (male as reference), age, CGAS change (change score between baseline and discharge), HoNOSCA change (change score between baseline and discharge) and parental satisfaction using regression models. Due to the small sample size, we only performed univariable analyses to avoid multiple hypothesis testing (64, 65). In addition, and considering that not all patients received medication treatment, we performed a secondary analysis exploring perceived helpfulness using all other 5 items comparing parental responses of children receiving and not receiving medication during inpatient status.

The cut off for statistical significance was established at *p* < 0.05 and the confidence interval are reported. All analyses were performed in STATA 14.0. All the analyses were conducted using complete data.

## Results

### Sample Description

A total of 73 patients were included in this study (48%, females). Most of patients were White (72.6%), and the mean age at admission was 123.8 months (*SD* = 18.2). The average length of admission was 153 days (*SD* = 93). Most of the patients were receiving medication during their inpatient treatment (83.6%). A 53.4% of children did not live with two biological parents (ICD 10: anomalous parenting).

Regarding the mean CGAS score, it was 30.8 (*SD* = 11.3) at admission and 57.8 (*SD* = 14.3) at discharge. The mean HoNOSCA score was 20.8 (*SD* = 6.7) at admission and 11.3 (*SD* = 6.5) at discharge.

Regarding the WHQ, 52 (71.2%) carers answer all or some of the questions, while the ASQ was answered by 56 (76.6%) carers.

### Psychometrics Properties of the WHQ

Table 2 shows the descriptive statistics of all six items and loading factors. The EFA found one factor with eigenvalue over 1, which explained 91% of the variance (Table 3). All items had a loading factor over the cut off used as reference.

**Table 2:** Descriptives and standardized factor loadings for What was Helpful Questionnaire (WHQ) items.

**Items**	**Mn**	**SD**	**Var**	**Skew**	**Kurt**	**w**
Q1. Family work	4.36	0.78	0.60	−0.98	3.22	0.75
Q2. Work on parenting skills	3.77	0.91	0.84	−0.73	3.58	0.77
Q3. Behavioral work	4.31	0.84	0.70	−1.05	3.38	0.81
Q4. Individual psychological work	4.29	0.81	0.65	−0.81	2.71	0.70
Q5. Medication	4.31	0.76	0.58	−0.86	3.16	0.38
Q6. Care plans	4.31	0.84	0.70	−1.67	6.82	0.59
Total scale	4.22	0.56	0.31	−0.53	2.37	

*Md, median; SD, standard deviation; skew, skewness; kurt, kurtosis; w, weights or factor loadings*.

**Table 3:** Exploratory Factor analyses of the items of the What was Helpful Questionnaire (WHQ).

**Factor**	**Eigenvalue**	**Difference**	**Proportion**	**Cumulative**
Factor1	2.79345	2.37947	0.9148	0.9148
Factor2	0.41397	0.13933	0.1356	1.0504
Factor3	0.27464	0.30749	0.0899	1.1403
Factor4	−0.03285	0.10049	−0.0108	1.1295
Factor5	−0.13334	0.1289	−0.0437	1.0859
Factor6	−0.26225		−0.0859	1

Table 4 shows the reliability of the scale WHQ. The item-test correlation scores were high for all items, with the exception of the item Medication (0.48). The scale has satisfactory internal consistency (0.77). Calculating the Cronbach's alpha of the scale without the item Medication and using the data of parents of children who did not receive medication, the alpha for the total scale was similar (0.77), however the item-test correlation score of the item Medication improved (0.50).

**Table 4:** Reliability of the What was Helpful Questionnaire (WHQ).

**Items**	**Item-test correlation**	**Item-rest correlation**	**Avarage interitem covariance**	**Alpha**
Q1. Family work	0.75	0.61	0.23	0.71
Q2. Work on parenting skills	0.75	0.59	0.22	0.72
Q3. Behavioral work	0.81	0.68	0.21	0.69
Q4. Individual psychological work	0.71	0.54	0.24	0.73
Q5. Medication	0.48	0.25	0.31	0.80
Q6. Care plans	0.61	0.41	0.26	0.76
Total scale			0.24	0.77

### Association Analysis

No associations were identified between WHQ total score and age, gender, CGAS change or HoNOSCA change. Considering the mean value of the scale without the medication item (q5) we found that the parents of children receiving medication had a mean score of 4.2 (*SD* = 0.6), which was not statistically different from the mean score reported from parents of children not receiving medication (4.1, *SD* = 0.7).

The only strong relationship found was between the WHQ total score and parental ASQ total score was found (β = 0.65, *p* = 0.000) (See Table 5).

**Table 5:** Results of the associations analyses.

**Variable**	**Beta**	***P*-value**	**95% CI**	
Age	0.001	0.789	−0.008	0.010
Gender (reference Male)	−0.002	0.991	−0.315	0.312
CGAS change	0.0001	0.965	−0.009	0.009
HoNOSCA change	−0.003	0.836	−0.027	0.022
Parental satisfaction	0.654	0.000	0.435	0.873

## Discussion

To the authors' knowledge, this is the first study adding evidence for the validity of a scale of perceived helpfulness for children receiving treatment in a mental health inpatient setting. We have found that the WHQ is an easy-to-use tool, with satisfactory psychometric properties, and that the construct of perceived helpfulness measured by this scale is unidimensional, where most items had high factor loadings.

Knowing that the impact of serious mental health conditions in children and their families can be severe and prolonged, it is important to comprehensively assess, not only the specific needs of care (psychological and pharmacological treatment), but also the perceived helpfulness of different aspects of care.

All patients received treatments and services included in the scale, with the exception of medication. A 16% of children did not receive medication, therefore, parents could not respond to this item. We suggest to re-arrange the order of the items in the scale, putting the item “Medication” at the end and after the statement: “If your child had received any medication on the ward, you consider that it was helpful” answering with the same Likert scale. A further evaluation of this amended scale would be warranted.

Each of the treatments provided by the unit is extensively explained to the parents and children, including information about the objective of the intervention, efficacy, potential benefits and risks. Other authors have shown that the beliefs of general public greatly influence the acceptance and perceived helpfulness from different treatments (66). Therefore, providing complete information regarding each intervention implemented by health professionals is important to collaborate in the general care of patients, especially if some the interventions are locally implemented in a unit.

Among the limitations, we have to mention the small sample size, which does not provide sufficient power to conduct a robust exploration of the psychometric features of the questionnaire. This small sample size also had reduced the possibilities to perform other psychometric measures. For example, a confirmatory factor analysis to explore further the relationship between items and to assess theoretical measurement structure. We also cannot consider the association results as definitive because the small sample size only allows us to consider these results as exploratory. Additionally, we could not include other variables, such as the children's diagnosis and explore their associations with parental perceived helpfulness. We also did not ask information regarding demographic data from parents because we wanted to reduce the burden associated to answer a questionnaire too long considering the context of having an inpatient offspring for a long time. However, demographic information such as education, work activities and age may have helped to find differences on understanding and responding the WHQ.

Future research may include a larger sample and young people receiving treatment in the community or in an outpatient setting.

## Conclusions

Initiatives to assess parental perceived helpfulness of the treatments received by their children are needed to facilitate the identification of potential gaps between professionals' evaluation of proposed treatment plans and families' opinions about them. The measure designed and assessed in this study is a good candidate to reduce this gap, and it may also be implemented in other settings of care delivery.

Our results added evidence for the validity and reliability of the WHQ to measure parental perceived helpfulness of the multidimensional intervention offered in inpatient children's units. Administration of WHQ in larger independent samples will help to assess the psychometric features of this instrument, for example, testing further the dimensionally of the construct using confirmatory factor analyses; and may also confirm the generalizability of these findings and help clarify the relative contribution of specific aspects of care in perceived parental helpfulness.

## Ethics Statement

This project received ethical approval by the South London and Maudsley National Health Service (NHS) Foundation Trust Child and Adolescent Mental Health Services (CAMHS) Clinical Governance/Audit Committee.

## Author Contributions

MK and SF conceived and designed the study; IM collected the data; MK, SF, and OH supervised the collection of data; IM, JG, and MK analyzed and interpreted the data, and produced the drafting of the manuscripts. All authors provided a critical revision of the manuscript.

### Conflict of Interest Statement

The authors declare that the research was conducted in the absence of any commercial or financial relationships that could be construed as a potential conflict of interest.

## Abbreviations

UK: United Kingdom
WHQ: What was Helpful Questionnaire
SDQ: Strengths and Difficulties Questionnaire
CGAS: Children's Global Assessment Scale
HoNOSCA: Health of the Nation Outcome Scales for Children and Adolescents
ASD: Autism spectrum disorder
HD: Hyperkinetic Disorders
CBT: Cognitive Behavioral Therapy
OT: Occupational Therapy
LOS: Length Of Stay
ASQ: Acorn Satisfaction Questionnaire
SD: Standard Deviation
NHS: National Health Service
CAMHS: Child and Adolescent Mental Health Services
EFA: Exploratory Factor Analysis
ICD: International Classification of Diseases.
