# Supplementary material for: What Was Helpful Questionnaire (WHQ): Psychometric Properties of a Novel Tool Designed to Capture Parental Perceived Helpfulness of Interventions in Children Requiring Mental Health Inpatient Care
Source: Front Psychiatry. 2019 Feb 26;10:80. doi: 10.3389/fpsyt.2019.00080 (PMC6399118; doi:10.3389/fpsyt.2019.00080)
Supplement: Supplementary file 1 [file Data_Sheet_1.docx]

# Appendix: Instrument WHQ

WHAT WAS HELPFUL ON ACORN LODGE?

Child’s name: ……………………………………… Child’s age: …………

Family work

Strongly Disagree Neutral Agree Strongly

Disagree Agree

Work on parenting skills

Strongly Disagree Neutral Agree Strongly

Disagree Agree

Behavioural work for your child as part of the ward environment

Strongly Disagree Neutral Agree Strongly

Disagree Agree

Individual psychological work for your child

Strongly Disagree Neutral Agree Strongly

Disagree Agree

Medication

Strongly Disagree Neutral Agree Strongly

Disagree Agree

Care plans

Strongly Disagree Neutral Agree Strongly

Disagree Agree
